# Changes in Short- and Medium-Chain Fatty Acids and Sugars During Kombucha Fermentation of Tea and Coffee Byproducts and Their Relation to Sourness

**DOI:** 10.3390/foods15122074

**Published:** 2026-06-08

**Authors:** Amanda Luísa Sales, Marco Aurelio Dal Sasso, Débora de Almeida Azevedo, Alessandro Maia, Verônica Calado, Marco Antônio Lemos Miguel, Adriana Farah

**Affiliations:** 1 Núcleo de Pesquisa em Café Prof. Luiz Carlos Trugo (NUPECAFÉ), Laboratório de Química e Bioatividade de Alimentos, Instituto de Nutrição, Universidade Federal do Rio de Janeiro, Avenida Carlos Chagas Filho, 373, CCS, Bl. J, Rio de Janeiro 21941-902, Brazil; 2 Instituto de Química, Universidade Federal do Rio de Janeiro, Avenida Horácio Macedo, 1281, LADETEC, Polo de Química, Ilha do Fundão, Rio de Janeiro 21941-598, Brazil; 3 Laboratório de Termoanálises e de Reologia (LABTER), Escola de Química, Universidade Federal do Rio de Janeiro, Avenida Athos da Silveira Ramos, 149, CT, Cidade Universitária, Rio de Janeiro 21941-909, Brazil; 4 Laboratório de Microbiologia de Alimentos, Instituto de Microbiologia Paulo de Góes, Universidade Federal do Rio de Janeiro, Avenida Carlos Chagas Filho, 373, CCS, Bl. I, Rio de Janeiro 21941-902, Brazil

**Keywords:** organic acids, fermented beverages, coffee byproducts, acetic acid

## Abstract

Kombucha is traditionally produced by fermenting *Camellia sinensis* tea and sugar in a consortium of microorganisms called SCOBY (Symbiotic Culture Of Bacteria and Yeasts). Short- and medium-chain fatty acids and other organic acids in K are mainly produced by acetic acid bacteria, which contribute to the typical K taste. Coffee is one of the most widely consumed beverages in the world and one of the most traded commodities globally. Harvesting during coffee production generates tons of byproducts generally considered of low value, including cascara (CC), composed of dried pulp and skin, and leaves (CL). To date, few studies have investigated the production of short- and medium-chain fatty acids and monosaccharide’s profile during traditional kombucha fermentation, and their composition in kombuchas prepared from substrates other than *C. sinensis* is even scarcer. This study followed the changes in sugars and the production of short- and medium-chain fatty acids during K fermentation of black tea (BT), CC, and CL and associated their concentrations with physicochemical parameters (total soluble solids (TSS), pH, and titratable acidity (TA)) and the perceived acidity of the beverages evaluated by a trained panel and untrained consumers. BT K, a SCOBY, and 10% sucrose were added to infusions of arabica CC, CL, or BT. The mixture was fermented for 0, 3, 6, and 9 days. Organic acids were analyzed by GC-MS; sucrose and monosaccharides were analyzed by HPLC-RID. The Rate All That Apply (RATA) test was used for sensory analysis. Results were treated by ANOVA–Fisher and Pearson correlation tests with significance at *p* < 0.05. Glucose, fructose, arabinose, xylose, cellobiose and glycerol were identified in the infusions. On average, sucrose concentration decreased by 28% up to day 9, considering all K samples, accompanied by TSS decrease. Eight organic acids were semi-quantified, with acetic being the major acid in all beverages (8.4 to 1971 mg L^−1^) and isovaleric being the lead minor acid (0.7 to 17.7 mg L^−1^). Additional acids identified were: butanoic, 2-methylpropanoic, pentanoic, 3-methylpentanoic, hexanoic, and octanoic acids. TA values and sourness perceived by consumer assessors increased generally, even though in CC Ks, the acid concentration decreased by day 9. TA, sourness, and sparkling and fizzy mouthfeel correlated positively in all Ks. In general, although the total acid concentration was mainly higher on days 3 or 6, CO_2_ formation, among other organic acids, probably increased TA and sourness on day 9. Although it is generally accepted that pH and organic acid concentrations are directly associated with sour taste, it is not possible to accurately predict and modify sour taste intensity in kombucha based only on these parameters, given that other factors, such as the production of CO_2,_ the existence of buffer systems, and the presence of sugars and other soluble solids, will probably affect the perceived acidity and sourness.

## 1. Introduction

Fermented foods are defined as foods made through desired microbial growth and enzymatic conversions of food components. They accompanied and likely facilitated the transition from hunter-gatherer communities to sedentary agricultural communities in the Neolithic Revolution about 14,000 years ago. Despite their long history, popularity, and culinary importance, the acceleration and industrialization of food production over the past century have reduced the diversity of fermented foods, particularly in the West. Recently, however, fermented foods have regained popularity in Western diets that emphasize artisanal processes. One reason for this surge in interest is their health-promoting potential [1,2].

Kombucha is traditionally produced by fermenting *Camellia sinensis* tea and sugar in a consortium of microorganisms called SCOBY (Symbiotic Culture Of Bacteria and Yeasts). It originated in Asia around 220 B.C. and was first used for its health properties [3]. The traditional beverage can be prepared with both black and green teas, with black tea being more popular [4]. The global kombucha market size was estimated at USD 4.26 billion in 2024 and is expected to grow at a compound annual growth rate of 7.6% from 2025 to 2033 [5]. The chemical composition depends on the substrate used for kombucha production, but in general, it contains phenolic compounds, mono- and disaccharides, and organic acids, among other things [6].

Acidity is one of the most important organoleptic parameters in fermented beverages, such as wine, and also in coffee brews, mainly due to the presence of weak organic acids [7,8]. However, organic acids are not important just because of their organoleptic contribution. They also play an important biotechnological role in industrial fermentation. These acids are in equilibrium with their salts, acting as buffers and, thus, maintaining the pH of wines and kombuchas [7,9].

Organic acids in kombucha also contribute significantly to flavor. They are mainly produced by acetic acid bacteria, although the contribution of yeasts and lactic acid bacteria should not be neglected [10]. Flavor is a combination of taste, aroma, and mouthfeel attributes, defining a specific sensory profile that ultimately affects consumer judgment regarding the overall quality of some fruits. Taste largely depends on water-soluble compounds, such as soluble sugars (fructose, glucose, and sucrose) and organic acids, which confer sweetness and/or sourness sensations and thereby determine the organoleptic properties of fruits and foods in general [11,12]. Organic acids can contribute specific types and intensities of acidity in different ways, depending on their sensory characteristics, concentration in the beverage, and also on their strengths determined by their dissociation constant (Ka) value, which is used to describe a tendency of compounds or ions to dissociate [13]. In organic acids, the number of carbons is important to determine the sensory results. While medium- and long-chain organic acids tend to have bitter and unpleasant tastes, sometimes with a distinct fat flavor known as *oleogustus,* short-chain organic acids or short-chain fatty acids (SCFA), as they are also called, such as acetic acid and isovaleric acid, tend to taste sour [14,15]. Interactions with other components can also affect perceived acidity or sourness. For example, the perception of sourness is weakened by the addition of sugar; likewise, low sugar contents tend to increase the perception of sourness [16].

Coffee is one of the most widely consumed beverages in the world and one of the most traded commodities globally. It was estimated that worldwide coffee production in 2024/2025 was approximately 10.6 million tons. In Brazil, coffee production during this period represented about 31% of global coffee production [17]. Harvesting during coffee production generates tons of byproducts, such as cascara and leaves, which are often considered of little or low value compared to the highly valuable coffee seeds, especially in organic crops [18]. In Europe, coffee cascara (also called husk) and leaves are officially recognized as novel foods [19,20].

In our previous studies evaluating the sensory acceptance and characterization of kombuchas made with black tea, coffee, and coffee byproducts, an increase in sourness was perceived with fermentation [21,22]. To date, only a few studies have investigated the production of organic acids during traditional kombucha fermentation, especially organic acids other than acetic acid [23,24]. Moreover, reports on short- and medium-chain fatty acids in kombuchas prepared with different substrates from *C. sinensis* are even scarcer. This study aimed to identify the main organic acids and simple sugars produced and degraded during kombucha fermentation of infusions made with coffee byproducts and to tentatively associate their concentrations with physicochemical parameters and with the consumer assessors’ perception of acidity and sourness.

## 2. Materials and Methods

### 2.1. Samples

Leaves from the leading brand of commercial *Camelia sinensis* black tea (BT) were acquired in a market in Rio de Janeiro, Brazil; two samples of *Coffea arabica* cascara (CC) (one from dry processed fruits and one from wet processed fruits) were acquired directly from producers in Domingos Martins, Espírito Santo, Brazil (CC1), and from Matagalpa, Nicaragua (CC2), respectively.

Leaves from the leading commercial brand of dried, unfermented *C. arabica* leaf tea (CL) in the West, harvested and processed in Nicaragua and sold in Canada, were used.

### 2.2. Microbial Consortium, Infusions, and Kombuchas

Infusions of BT and CC were prepared at 3% (weight/volume—*w*/*v*), while those of CL were prepared at 2.5% (*w*/*v*), based on previous sensory results [25,26]. Water at 95 °C was poured over the raw material, letting it steep for 10 min, and the mixture was filtered using regular filter paper (Mellita^®^, Minden, Germany) for bulk tea. Samples were collected for chemical and physicochemical analysis. Prior to inoculation and incubation, all infusions were passed through a Millipore 0.22 µm filter (Millipore, Burlington, MA, USA) and all material was sterilized. Inoculation and manipulation of the working material wereperformed in a laminar flow hood.

*Kombucha Consortium and fermentation*: The Kombucha Consortium was part of the collection of the Microbiology Institute of the Federal University of Rio de Janeiro in Brazil. The SCOBY was mainly composed of the following acetic acid bacteria, *Komagateibacter rhaeticus* (40–65%), *K. europaeus* (18–35%), *K. intermedius* (0.3%), and *K. xylinus* (0.3%), and the following yeasts, *Pichia fermentans* and *Pichia kluyveri* (90–92%), *Brettanomyces bruxelensis* (6–7%), and *Saccharomyces cerevisiae* (0.5%), as well as other minor bacteria and yeasts described in Sales et al. [21,22,27]. All kombucha fermentations were performed at 23 °C.

Previously cultivated in green tea, the consortium was fermented 3 times separately in black tea, coffee cascara, and coffee leaf tea infusions before use in the experimental system to stabilize the microbial consortium in these matrices [24]. Kombucha beverages were prepared according to the protocol described by Nummer [28], as listed below.

*Black tea kombucha:* BT K was prepared by mixing 90% (*v*/*v*) black tea infusion, 10% (*v*/*v*) of BT K starter culture, 10% (*w*/*v*) table sugar (sucrose), and 2.5% (*v*/*v*) SCOBY. The mixture was allowed to ferment. Samples were collected before fermentation (d0) and after 3 (d3), 6 (d6), and 9 (d9) days of fermentation. An additional sample with a pH of 2.8 ± 0.05 was collected after 14 days of fermentation and used as a starter for coffee cascara and leaf kombucha production.

*Coffee cascara kombuchas*: CC Ks were prepared using 90% (*v*/*v*) of the CC infusion, 10% (*v*/*v*) of BT K starter culture, 10% (*w*/*v*) sugar, and 2.5% (*w*/*v*) of SCOBY. The mixture was allowed to ferment. Samples were collected before fermentation (d0) and after d3, d6, and d9 of fermentation.

*Coffee leaf kombucha*: CL Ks were prepared by mixing 90% (*v*/*v*) CL infusions, 10% (*w*/*v*) sugar, 10% (*v*/*v*) BT K starter culture, and 2.5% (*w*/*v*) of SCOBY and fermented. Sampling was done at d0, d3, d6 and d9.

### 2.3. pH, Total Titratable Acidity, and Total Soluble Solids Determination

pH was measured using a pH meter (Kasvi K39-0014PA, São José dos Pinhais, São Paulo, Brazil). Titratable acidity (TA) was determined in triplicate by titration with 0.1 N NaOH, using phenolphthalein as an indicator, according to the Adolfo Lutz Institute [29]. Results were expressed in mEq/L. Total soluble solids (TSS) were evaluated using a handheld refractometer (Pocket Refractometer Pal–1, ATAGO, Tokyo, Japan). Results were expressed in °Brix.

### 2.4. High-Performance Liquid Chromatography Analysis of Sugars

Prior to analysis, samples were diluted 10-fold and then filtered twice through a paper filter (Whatman nº1) and passed through a membrane with a 0.45 µm pore size (MF-Millipore, Merck, Darmstadt, Germany). Non-diluted plain (unsweetened) infusions of each beverage were used as controls. Sucrose (PROQUIMIOS Produtos Científicos, Rio de Janeiro, Brazil), minor mono- and disaccharides (glucose, fructose, arabinose, xylose, cellobiose) and the polyol glycerol (VETEC Química Fina, Rio de Janeiro, Brazil) were analyzed in triplicate according to Wischral et al. [30] with adaptations, using a High-Performance Liquid Chromatography–Refractive Index Detector (HPLC-RID) system (mod.# 2414,Waters, Milford, MA, USA), a Hi-Plex column H8 µm (300 × 7.7 mm; Agilent, Santa Clara, CA, USA) at 30 °C with 20 µL of injection volume, and H_2_SO_4_ 0.005 mol/L as a mobile phase at 0.4 mL min^−1^. For monosaccharides, the column temperature was 60 °C, and the mobile phase flow was 0.6 mL min^−1^. External standard curves were used for sugar identification and quantification.

### 2.5. Gas Chromatography–Mass Spectrometry Analysis of Organic Acids

Three grams of sodium chloride were added to 5 mL of each sample, and the mixture was homogenized for 10 s. After the extractor solvent, the deuterated acid standards were added, and the tubes were closed. The solution was agitated for 10 min in an orbital agitator (FANEM, mod. 255-B, São Paulo, Brazil) and centrifuged for 10 min at 4000 rpm. After separation, the organic phase was collected, leaving minimal solvent in the aqueous phase.

For this semi-quantitative analysis, the following internal standards (CDN Isotopes, Pointe-Claire, QC, Canada) were used at 1 mg mL^−1^ in methyl *terc*-butyl-ether: butanoic acid-D_11_ (99% purity) and octanoic acid-D_15_ (99% purity). The solvent methyl *terc*-butyl-ether was also used as a blank (control).

The analysis of organic acids in the infusions and kombuchas was performed by a gas chromatographer (Agilent Technologies, 6890N Palo Alto, CA, USA) coupled to a mass spectrometer (Agilent 5973) and equipped with an auto sampler (Agilent 7673), according to the method described by Dal Sasso [31] with adaptations, using a Carbowax column 100% Polyetylenglycol (30 m, 0.25 mm d.i., 0.25 μm df; Agilent Technologies, Palo Alto, CA, USA).

Chromatographic conditions were: 60 °C, 1 °C, heating rate 4 °C min ^−1^ until 120 °C, 2 °C, heating rate 10 °C min^−1^ until 220 °C with isothermal conditions for 15 min. The mass analyzer was set to 150 °C and the ion font to 230 °C. Extracts previously prepared were injected at 1 μL in splitless mode (1 min), and the temperature of the injector was 240 °C; capture gas flow was 1.2 mL min^−1^, and the solvent delay was 3.5 min; transference line temperature was 240 °C; mass scanning was performed from *m*/*z* 20 to 250. Ion source temperature was 230 °C. Analytes were tentatively identified using linear retention indexes (LRIs) and confirmed using the National Institute of Standards and Technology (NIST) library database [32]. The Agilent MSD ChemStation, version E, Rev. E.02.00 (Agilent Technologies, Santa Clara, CA, USA) software was used for data collection and processing. The compounds were identified based on their LRI and the mass spectra of the NIST library [32]. To improve the accuracy of compound identification, only substances with a match factor greater than 700 (70% similarity) were selected for data processing. Nevertheless, all match factors for organic acids were equal to or greater than 800. The deuterated internal standards—namely butanoic and octanoic—were used for semi-quantification of the identified organic acids at a concentration of 100.0 µg mL^−1^. Each deuterated acid was used to quantify a specific range of the identified acids. Deuterated butanoic acid was used from acetic acid to hexanoic acid and deuterated octanoic acid from 3-hexenoic acid to octanoic acid. The selection criterion was based on the retention time ranges in the chromatogram. The analyses were performed in triplicate.

### 2.6. Sensory Analysis (Rate All That Apply—RATA)

The Ethical Committee of Clementino Fraga Filho University Hospital at the Federal University of Rio de Janeiro (UFRJ) approved this study (approval # 4.513.606). The subjects, including students, teachers, visitors, and employees at the UFRJ Health Sciences and Technology Centers living in different areas of Rio de Janeiro, provided written consent after being thoroughly informed. Only the kombuchas made with coffee byproducts were selected for sensory analysis. The eligibility criteria for this study included habitual consumption of kombucha or sparkling beverages, such as sparkling water, ciders, and soft drinks. Individuals who had a positive COVID-19 diagnosis and experienced loss of taste and/or smell were excluded from the study, as were individuals with any other conditions that could affect smell and taste. A total of 216 participants took part in the RATA tests (performed as in Meilgard et al. [33]) after exclusions. Assessors were provided with a checklist built by a trained panel containing sensory descriptors related to aroma, flavor, and mouthfeel. This included acetic vinegar flavor/aroma, apple vinegar flavor/aroma, sour taste, sparkling mouthfeel, and fizzy mouthfeel. To evaluate the perceived intensities in the taste and flavor descriptors of the kombucha samples, assessors were asked to score the descriptor based on its intensity using RATA scores (1 = low intensity, 2 = medium intensity, and 3 = high intensity).

Demographic information such as gender, age, level of education, family income, and kombucha, soft drinks and sparkling beverages consumption was collected prior to sensory analysis. Approximately 30 mL of kombucha was presented at 4 °C in 40 mL acrylic cups with dimensions 43 × 56 mm, coded with three-digit random numbers, and distributed in a balanced way to avoid the consistent influence of neighboring samples on sensory perception. Crackers and spring water at room temperature were offered between samples to clean the palate.

### 2.7. Statistics

Analysis of variance (ANOVA) followed by Fisher’s test (GraphPad Prism, version 8.4.2, Informer Technologies, Los Angeles, CA, USA) was used to compare the contents of sugars and organic acids, as well as the physicochemical parameters, among samples. Pearson’s correlation (Statistica^®^, version 14, Palo Alto, CA, USA) was used to examine relationships among total acid concentration, physicochemical parameters, and sensory responses. Differences were considered significant at *p* ≤ 0.05.

## 3. Results and Discussion

### 3.1. Physicochemical Parameters

**Figure 1:**
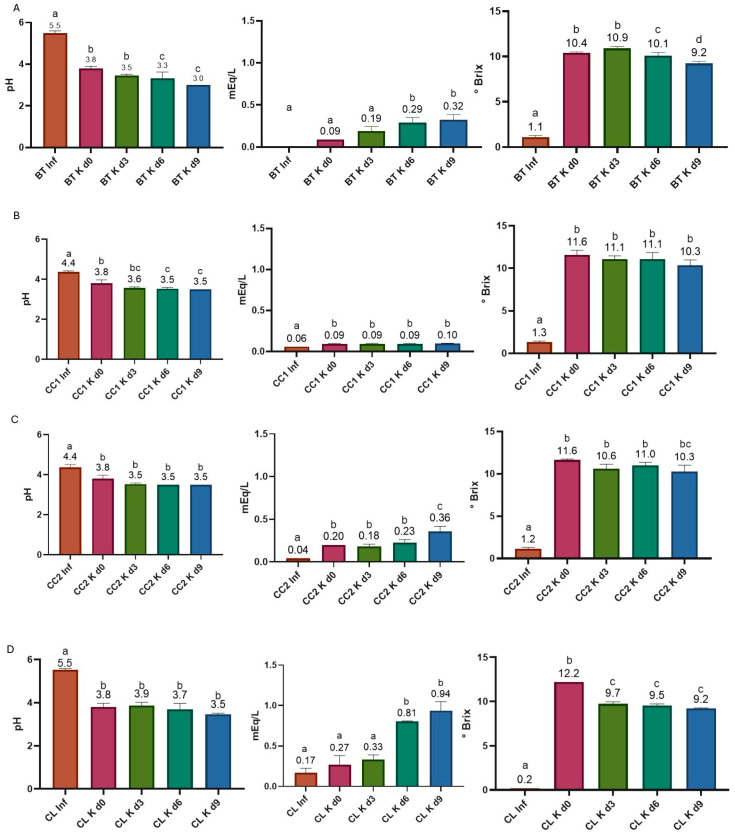
Physicochemical parameters (from left to right: pH, titratable acidity, and total soluble solids) in black tea (**A**), coffee cascara 1 (**B**), coffee cascara 2 (**C**), and coffee leaf (**D**) beverages. Data are expressed as the mean ± standard deviation for triplicate analyses; different letters for the same beverage (on the same line) indicate significant differences (*p* ≤ 0.05); BT: black tea; CC1: coffee cascara 1; CC2: coffee cascara 2; CL: coffee leaf; inf: infusion; K: kombucha; d0, d3, d6, d9: 0, 3, 6, 9 days of fermentation.

The physicochemical parameters (pH, TA and TSS) of BT, CC, and CL infs and K are presented in Figure 1. pH values of BT infs were similar to CL infs, while CC infs showed lower pH and higher TA, which agrees with values observed in DePaula et al. [25,26]. Fermentation caused a mild decrease in pH and an increase in TA, as expected, although changes in pH were not as dramatic as in TA, with weak correlations between both in BTK (r = −0.7381, *p* = 0.06) and CL K (r = −0.6493, *p* = 0.02).

BT K showed the largest change in pH during fermentation compared to other beverages, although TA increased more dramatically in CL K. TA plays an important role in the stability, color, taste, and aroma of fermented beverages like wine and kombucha [7]. Regarding CC Ks, TA values were stable in CC1 K, whereas a slight increase was observed in CC2 K during fermentation. The pH values obtained are within the range considered safe for human consumption (about 2.5 to 4.2–4.6) [28,34,35]. Kombuchas with a pH below 2.5 tend to have high acetic acid concentration, posing a risk to consumers’ health. Likewise, values above 4.2–4.6 may affect the beverage’s microbiological safety and the shelf life of these foods [34,35]. Similar results to ours were previously obtained for kombuchas from other new raw materials and kombucha-like beverages, such as acerola (*Malpighia emarginata*) [36] and kombuchas flavored with pitanga (*Eugenia uniflora* L.), umbu-cajá (*Spondia tuberosa)* fruit pulps [37] and red grape juice (*Vitis vinifera*) [38].

In relation to the mild change in pH and the weak negative correlations with TA, it is worth noting that, as with wine, a buffer system tends to be formed during kombucha fermentation due to the presence of the organic acids, and buffering in acidified foods is important in maintaining pH below 4.2–4.6, as aforementioned. The difficulty of measuring the pH of buffered solutions increases as the number of weak acids or bases in the solution increases, particularly if polyprotic acids like citric and phosphoric acids are present in mixed acid solutions, which is probably not the case in this study. Many acidic foods, including dressings and fermented and acidified vegetables, contain multiple low-acidity ingredients added to the final product, each with an undefined buffer capacity [39]. Also, according to Batali et al. [40], such a lack of sensitivity of pH compared to TA is chemically plausible because pH is a measurement of the dissociated hydrogen ion concentration, whereas TA is a measurement of acidic protons. TA, therefore, should be a more accurate measure of organic acids, which do not dissociate fully.

In relation to TSS, values in BT and CL beverages were similar, with significant changes during fermentation, while changes in CC beverages were not significant, suggesting a preference of the SCOBY culture for BT and CL compared to CC as substrates. In CL K, TSS strongly correlated positively with total sugars (r = 0.9190, *p* = 0.01) and negatively with TA (r = − 0.7105, *p* = 0.01). The same correlations occurred in BT K but were weaker (r = 0.6446, *p*= 0.02 and r = −0.5772, *p*= 0.05, respectively). In CC K, TSS only correlated with total sugars (r = 0.6120, *p* = 0.01). TSS also correlated positively with pH in CC K (r = 0.4542, *p* = 0.03) and in BT K (r= 0.7399, *p* = 0.06).

The present results are in agreement with those observed by May et al. [41], Czarnowska-Kujawska et al. [42], Câmara et al. [43] and Alemayehu et al. [44]. Although TSS includes other soluble solids besides sugar, lower values were expected in the studied infusions, given that they were unsweetened [45]. With the addition of sugar and other components, soluble solids increased from infusions to d0. Then, during fermentation, bacteria and yeasts metabolized sucrose to monosaccharides and organic acids. Commonly, at the beginning of the process, the sugar concentration still inhibits microbial growth and, consequently, the fermentation rate is slow, with low acid production. As fermentation proceeds, the concentrations of sugar and TSS decrease consistently, while the concentration of organic acids increases, pH decreases, and TA increases.

### 3.2. Monosaccharides and Sucrose Content in Beverages Made with Black Tea and Coffee Byproducts

**Figure 2:**
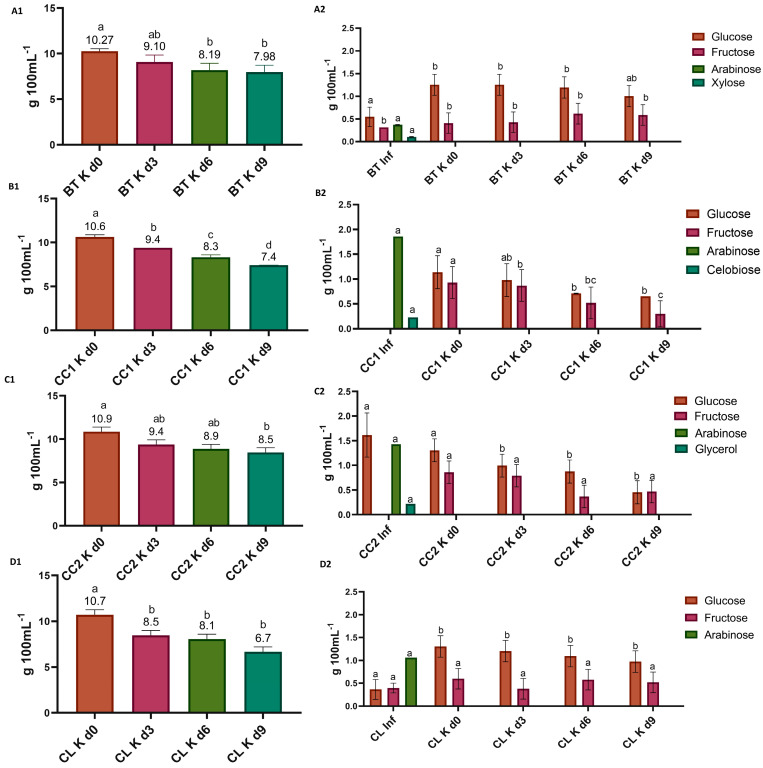
Contents of sucrose (left-1) and monosaccharides (right-2) (g 100 mL^−1^) in beverages prepared with: black tea (**A**), coffee cascara 1(**B**), coffee cascara 2 (**C**), coffee leaf (**D**) beverages. Data are expressed as the mean ± standard deviation for triplicate analyses; different letters on the same line for the same beverage indicate significant differences (*p* ≤ 0.05); BT: black tea; CC1: coffee cascara 1; CC2 coffee cascara 2; CL: coffee leaf; Inf: infusion; K: kombucha; d0, d3, d6, d9: 0, 3, 6, 9 days of fermentation.

The contents of monosaccharides and sucrose in the infusions and kombuchas made with black tea and coffee byproducts are presented in Figure 2.

Glucose, fructose and arabinose were identified in all infusions. Additionally, xylose was identified in the BT inf. All of these compounds have been previously identified in black tea infusions [46,47,48]. Glycerol was identified only in the fermented cascara (from the wet postharvest method).Glucose, fructose, and arabinose were identified in the CL inf. The monosaccharides glucose and arabinose were previously identified in coffee cascara and coffee leaves [49,50]. The presence of these compounds indicates the occurrence of type 2 arabinogalactan and galactomannan, which represent the soluble fiber fraction in coffee beans and other parts of the coffee plant [49,51]. Glycerol is one of the primary metabolites of ethanol fermentation by *Saccharomyces cerevisiae* and possibly other yeasts [52,53]. Its presence is consistent with fermentation during postharvest processing, before coffee and cascara were separated. Xylose and cellobiose have been previously identified in green coffee defatted cake [54], and xylose has also been identified in coffee parchment [55]. No significant difference was found in the contents of arabinose, xylose, glycerol, or cellobiose during kombucha fermentation.

A reduction in sucrose concentration from d0 to d9 of all Ks was observed (20% reduction in BT K, 25% on average in CC K, and 37% in CL K), with significant differences between fermentation days in general. In this process, yeasts and bacteria produce invertase, which cleaves the disaccharide sucrose to its monosaccharide components, glucose and fructose [41]. These are subsequently utilized in the production of ethanol, glycerol, and aliphatic acids, mainly acetic acid and CO_2_ [56,57]. The presence of glucose and fructose in black tea kombucha has also been observed by Villarreal-Soto et al. [24]. Higher sugar consumption in coffee leaf kombucha compared with black tea kombucha after 9 days of fermentation was also observed by Huang et al. [58]. According to the authors, this difference in sugar concentration may be attributed to variations in microbial profile or enzyme activity during fermentation due to the distinct substrates used.

### 3.3. Organic Acids

The main organic acids identified in our study are volatile acids, which play an important role in the flavor of fermented beverages [59]. The organic acids identified in the BT inf. and K are presented in Table 1.

No organic acids were detected in the BT inf, as expected, although acetic acid has previously been identified in black tea infusions by Chen et al. [62] and Zhang et al. [63]. Even though sourness is expected in kombuchas, in the black tea infusion, it negatively influences the sensory quality [10,64]. In BT K, the presence of organic acids increased as fermentation progressed until d6 and decayed in d9. It is known that low pH, among other factors, can restrict the growth of acetic acid bacteria [65]; therefore, the microorganisms in the consortium avoid high acid concentrations. One way to reduce acidity is the formation of volatile esters during fermentation [66].

Acetic acid was the main acid identified in BT K, with concentrations ranging from 86.7 to 188.4 mg L^−1^. This is the primary organic acid in kombucha, produced by acetic acid bacteria, and it is the primary contributor to kombucha sourness [67]. It is also found in black tea vinegar [62] and prune vinegar [68]. Acetic acid is formed at the beginning of fermentation. Acetic acid bacteria oxidize ethanol produced by yeasts during sugar fermentation to acetic acid under aerobic conditions and also produce other organic acids [62]. During this period, esters and alcohols can also be produced by yeasts [69].

2-Methylpropanoic acid was identified only in BT K d9. This is a volatile branched short-chain acid generated by the fermentation of branched amino acids, valine, leucine, and isoleucine, derived from proteins [70]. Butanoic acid was only identified in BT K d3 and d6. It has also been identified by Villarreal-Soto et al. [24] in black tea kombucha. It is found in other fermented foods such as beer and cherry (*Prunus cerasus* L.) vinegar [71,72].In fact, this acid and its derivatives have many applications in the production of chemicals, foods, pharmaceutical products, perfumes, and animal feed. Although butanoic acid itself has an unpleasant odor, its esters, such as methyl, ethyl, and amyl butyrate, are used as additives to enhance fruit fragrance and as aromatic compounds for the production of perfumes [73]. Also, butanoic acid has previously been identified in fermented milk and could be considered an odor-active compound, playing an important role in kombucha’s sour taste, together with acetic and hexanoic acid [74]. Considering its health effects, butanoic acid can be produced by colonic bacterial fermentation of non-starch polysaccharides [75]. Studies have demonstrated the beneficial effects of exogenous butanoic acid in chronic diseases, such as diabetes, obesity, and depression. It may also help prevent progression to renal failure and other adverse outcomes in patients with chronic kidney disease [76].

3-Methylbutanoic acid (isovaleric acid) was identified in BT Kd3 and d6. This acid has been identified in black tea infusions and kombucha [24,77,78]. It is produced by yeasts via the Ehrlich pathway and related pathways by converting the amino acid leucine into several metabolites until it is oxidized to isovaleric acid [79]. Isovaleric acid has been identified in honey [80] and beer [71]. 3-Methylpentanoic acid has previously been identified in wine and other alcoholic beverages, and its presence is associated with wine type and aging process [81].

Pentanoic acid (valeric acid) was identified in BT K d3 and d6. This acid is one of the short-chain acids produced by gut microbiota as a byproduct of the fermentation of dietary fiber [82]. Pentanoic acid is also present in *Valeriana officinalis*, a herb included in the pharmacopoeias of Europe and the United States, and can exert sedative effects, improve symptoms of insomnia, and reduce anxiety [82,83]. Additionally, 3-methylvaleric acid was identified in BT K d3 and d6. It is a product of amino acid metabolism that produces branched-chain SCFA [84].

Similar concentrations of hexanoic acid were identified in BT K d6 and d9. This organic acid is said to be an important compound in black tea [77], although we have not found it in our BT inf samples. This straight, six-carbon-chain organic acid has been previously identified in black tea kombucha by Leali et al. [78] and in fermented beverages such as beer [85]. It is also an important volatile active compound in cherry (*Prunus cerasus* L.) vinegar [72] and in sparkling wine, although it is not considered an odor-active compound [86]. In the food industry, caproic acid is an edible flavor compound and can be used as a food additive in butter and bread; through microbial fermentation, hexanoic acid can be produced with ethanol, acetic acid, glucose, D-galactitol and lactic acid as carbon sources [59].It can be produced by yeasts like *Saccharomyces cerevisiae* and anaerobic bacteria through several metabolic pathways until hexanoic acid formation [87,88,89]. Hexanoic acid contributes to the organoleptic properties of sake [90], and it is an odor-active compound in sparkling wine [86].

Octanoic acid was detected only in BT K d9 and has been reported in black tea kombucha [24,78]. It is a medium-chain acid with a wide range of applications in antimicrobials, surfactants, and cosmetics. It can also serve as a precursor for biofuels [91]. Considering fermented beverages, this organic acid is an important aroma compound in beer and sparkling wine [86,92].

#### Organic Acids in Beverages Made with Coffee Byproducts

The organic acids identified in the beverages made with coffee byproducts are presented in Table 2. Most of the identified acids were volatile compounds that contribute to the flavor of these beverages [93]. Generally speaking, like with BT K, the total organic acid concentrations peaked at d3 or d6 and decreased in d9.

Among the infusions, no organic acid was found in the CL inf nor in the CC1 inf. Acetic acid was only detected in CC2 samples, probably because CC2 was obtained from fruit fermentation during post-harvest processing [25], whereas CC1 was not fermented. Qin et al. [94] have identified acetic acid in unfermented cascara during post-harvest processing that could have been slightly fermented during natural drying or storage. DePaula et al. [95] have also identified it in dried *C. canephora* flowers.

The presence of acetic acid in all K d0 samples derives from the black tea kombucha (starter) added to the infusions to lower pH and initiate fermentation. Acetic acid was the main organic acid identified in all CC K (77.1 to 749.2 mg L^−1^) and CL K (46.5 to 1971 mgL^−1^) samples.

3-Methylpropanoic, butanoic, and 3-methylpentanoic acids were identified in CL K. These acids are known as short-chain acids [84]. In CC2, butanoic acid was only identified later on in d6. In CL K, its concentration increased by 105% from d6 to d9. In coffee, this acid could impart undesirable off-flavors according to Haile and Kang [96]. Also, butanoic acid has previously been identified in fermented milk and could be considered an odor-active compound, playing an important role in kombucha’s sour taste, together with acetic and hexanoic acids [74].

Pentanoic acid’s concentration increased up to CC1 K d6 and decreased in d9. This acid has been identified by Pua et al. [97] in a wet-processed coffee cascara and by DePaula et al. [95] in dried coffee flowers. 3-Methylpentanoic acid was identified in CL K d0, probably because it was present in the starter fraction.

3-Methylbutanoic and hexanoic acids were also identified in the byproduct kombuchas. 3-Methylbutanoic acid was the second-most prevalent among the identified acids. It has also been identified in coffee cascara [97] and dried coffee flowers [95], while hexanoic acid has been identified in dried coffee leaves [26].

In CC K beverages, octanoic acid was detected only in CC2 K d6, whereas in CL K it was detected from d3 to d9, suggesting dynamic changes in the fermentation process. Pua et al. [97] have identified this acid in coffee cascara. This is an important volatile compound in cherry (*Prunus cerasus* L.) vinegar flavor [72].

Although the total concentration of acids tended to increase from d0 tod3 in all kombucha samples, different behaviors were observed from d3 to d9. In CC1 K, the total acid concentration increased from d3 to d6 (144%) and decreased in d9 (43%), while in CC2 K, the total acid concentration peaked in d3 and decreased in d6 (38%) and from d6 to d9 (3%). In CL K, total acid concentrations peaked in d3, decreased in d6 (89%), and increased again in K d9 (99%), suggesting dynamic metabolic changes during fermentation. For example, acetic, butanoic, 3-methylbutanoic, and hexanoic acids are probable substrates for the formation of ethyl esters [98], contributing to floral aroma notes. The highest variation in total acids occurred in CC2 K, with higher concentrations than CC1 throughout fermentation. Despite these fluctuations, the physicochemical parameters did not show significant differences most of the time, probably due to the buffer capacity of the acids, as aforementioned.

### 3.4. Sourness Perception in Coffee Byproducts Kombuchas

**Figure 3:**
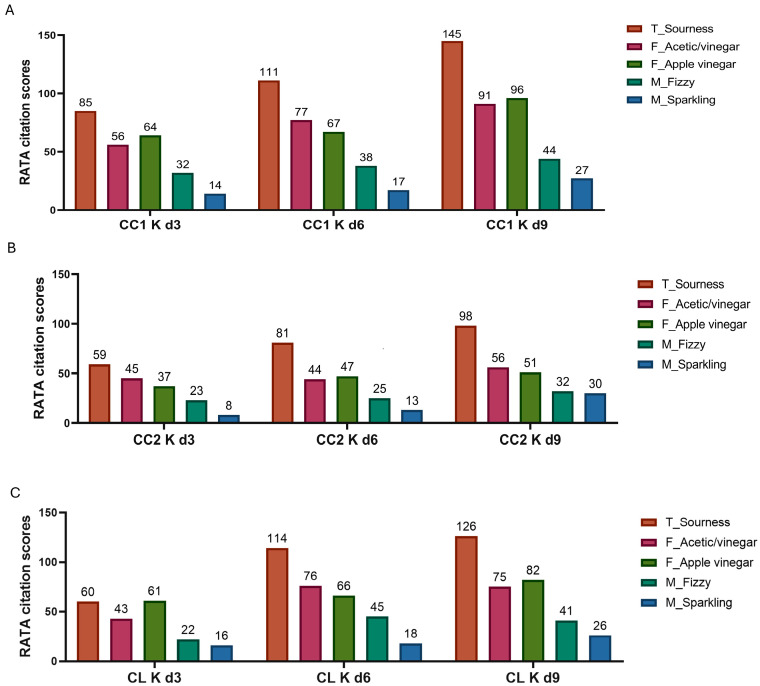
Rate All That Apply (RATA) citation scores (n = 216) for sour taste, acetic/vinegar flavor and apple vinegar flavor in (**A**) coffee cascara kombucha 1 (CC1 K), (**B**) coffee cascara kombucha 2 (CC2 K), and (**C**) coffee leaf kombucha (CL K), after 3, 6 and 9 days of fermentation. Note: T: taste; F: flavor; M: mouthfeel. CC1: coffee cascara from Brazil; CC2: coffee cascara from Nicaragua; CL: coffee leaf; Inf: infusion; K: kombucha; d0, d3, d6, d9: days 0, 3, 6, 9 of fermentation.

The RATA citation scores for the sensorial parameters of sour taste, acetic/vinegar flavor, apple vinegar flavor, sparkling mouthfeel and fizzy mouthfeel obtained from 216 untrained consumer assessors are shown in Figure 3. Sixty-five percent of the assessorswere female and 35% were male; they were aged 18–59 years old; 66% knew and appreciated kombucha but only 13% were regular consumers (it is a high-priced gourmet beverage in Brazil); and 35% regularly consumed sparkling beverages and soft drinks such as sparkling water, soda, apple juice, tonic water, sparkling wine and cider.

Even though our kombuchas were not artificially carbonated, both the trained panel who prepared the RATA list of attributes and the untrained consumer assessors were able to perceive, as fermentation progressed, growing sparkling and fizzy attributes, which strongly correlated with fermentation time (r = 0.9028, *p* = 0.000 for leaves and r = 0.9884, *p* = 0.000 for cascara). Both attributes strongly correlated with sourness (r = 0.9490 and r = 0.9028, *p* = 0.000 for leaves; r = 0.8982 and r = 0.9884, *p* = 0.000 for cascara). In these samples, the attributes apple vinegar and acetic vinegar were also strongly correlated with sourness, as expected (r = 0.9380 and r =0.9927, *p* = 0.000 for leaves; r = 0.9821 and 0.9826, *p* = 0.000 for cascara) (Figure 3). Similar correlations were observed between sparking/fizzy and apple/acetic vinegar attributes.

As expected, sourness correlated strongly with TA, with r > 0.90 and *p* = 0.000 for all samples. The typical sourness perception in kombucha is related to the production of organic acids throughout fermentation. Vinegar notes are also characteristic attributes of kombucha [99], although long fermentation tends to result in excessive sourness and vinegar notes, which can negatively impact overall liking [100] and acceptability [22,38,101].

As aforementioned, the total concentration of acetic acid and total organic acids peaked at d3 or d6. Nevertheless, the perception of sourness and related attributes by consumer assessors was lowest in d3 samples and peaked at d9. This could perhaps be explained by the production of other weak organic acids by acetic acid bacteria, such as gluconic acid, glucoronic acid, and citric acid [102], which were not analyzed in this study. However, considering that all other acids produced by acetic acid bacteria peaked earlier than d9, there would probably not be a plausible reason for higher production of gluconic and glucoronic acids later in kombucha fermentation. Also, although citric acid is produced by yeasts, it is a minor acid in kombucha and could not have caused such a strong sour sensation.

Taking into account that acetic acid is by far the major acid in kombuchas, and that its perception threshold is quite low, there are other facts that could explain the peaked sourness at d9. The first reason is the higher concentration of sucrose in the earlier fermentation days (K d3 followed by d6) compared to K d9, which was markedly felt by the trained assessors. The balance between sugar concentration and pH is critical in defining the flavor profile, as a higher sugar concentration can offset acidity and enhance palatability [103]. This was supported by the higher concentration of TSS in d3/d6, which is not only an important indicator of sweetness but also of thickness, viscosity and flavor intensity in kombucha and strongly influences sensory perception and consumer acceptance of beverages in general. According to Chen et al. [104], as the thickness/viscosity decreases, the sourness perception increases in fluid systems. This was corroborated by a strong negative correlation between TSS and sourness (r = −0.9188, *p* = 0.000 in CL K) and between total sugars and sourness (r = −0.9066, *p* = 0.000) in the present study.

The second reason for higher sourness perception in d9 would be the higher natural production of CO_2_ at this point, although most untrained consumer assessors (and not the trained assessors) considered the beverage to cause moderate fizzy and sparkly sensations. This was probably because they were comparing this experience with their previous experiences with artificially gasified beverages. It is known that CO_2_ tends to increase the acidity and sourness in beverages [105]. The increase in acidity was confirmed in our laboratory, when 1kgF/cm^2^ CO_2_ was progressively added to 0.1 L Milli-Q water (Millipore Sigma, Burlington, MA, USA) (pH 6.2) for 10, 20 and 30 min. The pH dropped to 5.1, 5.1 and 4.8, respectively, and TA increased from 0.25 mEq/L in Milli-Q water to 1.00 mEq/L for all periods of exposure to CO_2._ Therefore, this also justifies the fact that although the concentration of organic acids peaked at d3 or d6, TA peaked at d9, in association with sourness sensation and acetic acid-related sensory attributes. Nevertheless, the quantification of all parameters evaluated in this study, together with other organic carboxylic acids, would be ideal in a future study.

## 4. Conclusions and General Remarks

In this study, the concentration and role of total short- and medium-chain organic acids, sugars and CO_2_ in sourness and related sensory attributes in kombucha were explored. The concentrations of eight organic acids, one disaccharide, six monosaccharides, and one polyol were evaluated in black tea and coffee byproduct kombuchas. Acetic acid was the major acid in all samples, followed by minor additional acids, among which isovaleric acid was the most abundant. Although the concentration of organic acids peaked at earlier stages, the perception of sourness and acetic acid-related attributes increased continuously, mostly due to the decrease in sugars and TSS, increase in fluidity and increased CO_2_ production, which was reflected in the TA results and in the perception of fizzy and sparkling mouthfeel attributes. Therefore, although it is generally accepted that pH and organic acid concentrations are directly associated with sour taste, it is not possible to accurately predict and modify sour taste intensity in kombucha based only on these parameters, given that other factors, such as the production of CO_2,_ the existence of buffer systems, and the presence of sugars and other soluble solids, will probably affect the perceived acidity and sourness.

Despite the importance of acetic acid as the major contributor to the sourness and flavor of kombuchas, in future studies, the role and contribution of each of the identified organic acids present in the beverage to sourness and flavor should be explored, given that the same compound can produce different sensory results depending on its concentration, interaction with other compounds, and the sensory perception threshold in that matrix (for example, K d9 samples contained higher concentrations of isovaleric acid and caproic acids compared to other samples, and the resulting consequences should be evaluated). Additionally, as aforementioned, despite the major importance of acetic acid to acidity and sourness in kombuchas, the sourness contribution of other organic acids, such as gluconic, glucoronic, and citric acids, which were not analyzed in this study, cannot be ignored and should also be investigated.

Another important point to consider is that the perception of sourness largely depends on the individual’s taste sensitivity and salivary buffering capacity [106]. In our study, untrained assessors identified different levels of acidity/sourness in kombuchas during fermentation. The sour taste is mainly triggered by the detection of free hydrogen ions by taste-receptor cells assembled into taste buds, which are distributed across different papillae of the tongue and palate. Although pH/TA are important parameters, in addition to measurable acidity, the intensity perceived by the taster is affected by many factors, such as speed of taste bud penetration; the buffer capacity of acids; the molar concentration, which can cause variation in buffer pH by one unit; the strength of an acid; and its degree of dissociation [107]. Carbonation and soluble solid concentrations are additional factors, as aforementioned, although they may produce a delayed onset of perception [108].

The authors hope that the results obtained in the present study will contribute to the understanding of the dynamics between the chemistry and the sensory perceptions of kombucha, as well as the influence of the different raw materials in fermentation and sensory results.

## Figures and Tables

**Table 1:** Organic acid concentrations (mg L^−1^) in black tea infusion and kombuchas.

Organic Acid (Popular Name)	IUPAC Name ^A^	Structure ^A,B^	CAS#	BT Inf	BT K d0	BT K d3	BT K d6	BT K d9
Acetic acid	Ethanoic acid	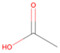	64-19-7	Nd	86.7 ± 2.3^a^	105 ± 3.2^b^	188.4 ± 1.6^c^	130.6 ± 2.0^d^
Isobutyric acid	2-Methylpropanoicacid	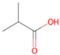	79-31-2	Nd	Nd	1.0 ± 0.0^a^	0.88 ± 0.0^b^	2.00 ± 0.0^c^
Butyric acid	Butanoic acid	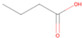	107-92-6	Nd	Nd	0.38 ± 0.0^a^	9.8 ± 0.0^b^	Nd
Isovaleric acid	3-Methylbutanoic acid	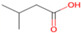	503-74-2	Nd	0.66 ± 0.08^a^	0.88 ± 0.1^a^	2.62 ± 0.0^b^	1.80 ± 0.0^b^
Valeric acid	Pentanoic acid	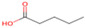	109-52-4	Nd	Nd	0.57 ± 0.0^a^	0.92 ± 0.2^b^	0.82 ± 0.0^c^
3-Methylvaleric acid	3-Methylpentanoic acid	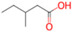	105-43-1	Nd	Nd	0.73 ± 0.0^a^	2.19 ± 0.0^b^	Nd
Caproic acid	Hexanoic acid	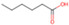	142-62-1	Nd	Nd	Nd	0.8 ± 0.0^a^	0.79 ± 0.0^a^
Caprylic acid	Octanoic acid	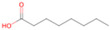	124-07-2	Nd	Nd	Nd	Nd	0.39 ± 0.2^a^
Total organic acids				Nd	87.4 ± 2.4^a^	108.6 ± 3.3^b^	205.6 ± 1.8^c^	136.4 ± 2.3^d^

Data are expressed as the mean ± standard deviation for triplicate analyses; Nd: not detected in the sample (^A^: PubChem (https://pubchem.ncbi.nlm.nih.gov/) [60]; ^B^: ChemSpider, Royal Society of Chemistry (https://www.chemspider.com/, accessed on 15 January 2026) [61]; CAS# (Chemical Abstracts Service) Registry Number, available in the NIST database [32]. Note: BT: black tea; Inf: infusion; K: kombucha; d0, d3, d6, d9: 0, 3, 6, 9 days of fermentation. Different lowercase letters in the same row indicate significant differences (*p* ≤ 0.05).

**Table 2:** Organic acid concentrations (mg L^−1^) in infusions and kombuchas made with coffee byproducts.

Organic Acid (Popular Name)	IUPAC Name ^A^	Structure ^A,B^	CAS#	CC1					CC2					CL				
				Inf	K d0	K d3	K d6	K d9	Inf	K d0	K d3	K d6	K d9	Inf	K d0	K d3	K d6	K d9
Acetic acid	Ethanoic acid	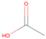	64-19-7	Nd	77.1 ±2.1^a^	172.5 ±4.5^b^	414.6 ±2.1^c^	207.9 ±7.9^d^	8.42 ±0.3^a^	207.0 ±0.6^b^	749.2 ±0.04^c^	323.7 ±8.8^d^	519.3 ±9.16^e^	Nd	46.5 ±0.9^a^	1971 ±2.0^b^	221.9 ±0.4^c^	438.1 ±0.6^d^
Isobutyric acid	2-Methylpropanoic acid	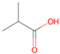	79-31-2	Nd	0.6 ±0.0^a^	1.9 ±0.0^b^	6.0 ±0.0^c^	3.1 ±0.2^d^	Nd	Nd	Nd	1.90 ±0.08^a^	Nd	Nd	Nd	Nd	1.80 ±0.0^a^	3.7 ±0.1^b^
Butanoic acid	Butanoic acid	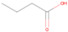	107-92-6	Nd	Nd	Nd	Nd	Nd	Nd	Nd	Nd	216.3 ±0.0^a^	Nd	Nd	Nd	Nd	Nd	0.8 ±0.1^a^
Isovaleric acid	3-Methylbutanoic acid	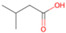	503-74-2	Nd	2.8 ± 0.2^a^	0.8 ±0.2^b^	3.6 ±0.10^c^	2.2 ±0.1^d^	Nd	1.48 ±0.19^a^	3.7 ±0.29^b^	Nd	3.19 ±0.20^b^	Nd	0.91 ±0.0^a^	17.7 ±0.7^b^	2.0 ±0.1^c^	3.5 ±0.4^d^
Valeric acid	Pentanoic acid	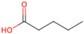	109-52-4	Nd	0.4 ±0.1^a^	Nd	0.67 ±0.0^b^	0.52 ±0.0^c^	Nd	Nd	Nd	Nd	1.46 ±0.00^a^	Nd	0.49 ±0.0^a^	4.1 ±2.4^b^	Nd	2.0 ±0.0^c^
3-Methylvaleric acid	3-Methylpentanoic acid	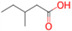	105-43-1	Nd	Nd	Nd	1.77 ±0.0^a^	0.5 ±0.0^b^	Nd	Nd	Nd	Nd	Nd	Nd	1.0 ±0.0^a^	Nd	Nd	Nd
Caproic acid	Hexanoic acid	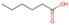	142-62-1	Nd	0.33 ±0.0^a^	Nd	0.3 ±0.0^a^	0.83 ±0.0^b^	Nd	0.99 ±0.0^ab^	Nd	0.65 ±0.38^a^	1.26 ±0.05^ab^	Nd	0.7 ±0.5^a^	2.15 ±0.0^b^	0.9 ±0.2^c^	2.0 ±0.1^b^
Caprylic Acid	Octanoic acid	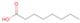	124-07-2	Nd	Nd	Nd	Nd	Nd	Nd	Nd	Nd	0.67 ±0.39^a^	Nd	Nd	Nd	109.4 ±0.0^a^	0.3± 0.04^b^	0.6± 0.3^b^
Total organic acids				Nd	81.2 ±2.4^a^	175.2 ±4.7^b^	427.0± 2.2^c^	214.0 ±7.9^d^	8.42 ±0.3^a^	209.5 ±7.9^b^	752.9 ±0.33^c^	543.2 ±9.6^d^	525.2 ±9.4^d^	Nd	49.6 ±1.4^a^	2104.4 ±5.1^b^	226.9 ±0.7^c^	450.7 ±1.6^d^

Data are expressed as mean ± standard deviation for triplicate analyses; Nd: non-detected in the sample; ^A^: PubChem (https://pubchem.ncbi.nlm.nih.gov/, accessed on 15 January 2026) [60]; ^B^: ChemSpider, Royal Society of Chemistry (https://www.chemspider.com/, accessed on 15 January 2026) [61]; CAS# (Chemical Abstracts Service) Registry Number, available in the NIST database [32]. Note: CC1: coffee cascara from Brazil; CC2: coffee cascara from Nicaragua; CL: coffee leaf; Inf: infusion; K: kombucha; d0, d3, d6, d9: days 0, 3, 6, 9 of fermentation. Different lowercase letters on the same row indicate significant differences (*p* ≤ 0.05).

## Data Availability

The original contributions presented in the study are included in the article, further inquiries can be directed to the corresponding author.

## Abbreviations

The following abbreviations are used in this manuscript:

SCOBY	Symbiotic Culture of Bacteria and Yeasts
BT	Black tea
CC	Coffee cascara
CL	Coffee leaf
Inf	Infusion
K	Kombucha
RATA	Rate All That Apply
TA	Titratable acidity
TSS	Total soluble solids
